# Rational Identification of Enoxacin as a Novel V-ATPase-Directed Osteoclast Inhibitor

**DOI:** 10.2174/138920312800493151

**Published:** 2012-03

**Authors:** Edgardo J Toro, David A Ostrov, Thomas J Wronski, L Shannon Holliday

**Affiliations:** 1 Department of Orthodontics, University of Florida College of Dentistry, Gainesville, FL 32610; 2 Department of Pathology, Immunology and Laboratory Medicine, University of Florida College of Medicine, Gainesville, FL 32610, USA; 3 Department of Physiological Sciences, University of Florida, Gainesville, FL 32610, USA; 4 Department of Anatomy & Cell Biology, University of Florida College of Medicine, Gainesville, FL 32610

**Keywords:** Osteoclast, vacuolar ATPase, actin, microfilaments, enoxacin, cancer, *Candida*.

## Abstract

Binding between vacuolar H^+^-ATPases (V-ATPases) and microfilaments is mediated by an actin binding domain in the B-subunit. Both isoforms of mammalian B-subunit bind microfilaments with high affinity. A similar actin-binding activity has been demonstrated in the B-subunit of yeast. A conserved “profilin-like” domain in the B-subunit mediates this actin-binding activity, named due to its sequence and structural similarity to an actin-binding surface of the canonical actin binding protein *profilin*. Subtle mutations in the “profilin-like” domain eliminate actin binding activity without disrupting the ability of the altered protein to associate with the other subunits of V-ATPase to form a functional proton pump. Analysis of these mutated B-subunits suggests that the actin-binding activity is not required for the “housekeeping” functions of V-ATPases, but is important for certain specialized roles. In osteoclasts, the actin-binding activity is required for transport of V-ATPases to the plasma membrane, a prerequisite for bone resorption. A virtual screen led to the identification of enoxacin as a small molecule that bound to the actin-binding surface of the B2-subunit and competitively inhibited B2-subunit and actin interaction. Enoxacin disrupted osteoclastic bone resorption *in vitro*, but did not affect osteoblast formation or mineralization. Recently, enoxacin was identified as an inhibitor of the virulence of *Candida* *albicans *and more importantly of cancer growth and metastasis. Efforts are underway to determine the mechanisms by which enoxacin and other small molecule inhibitors of B2 and microfilament binding interaction selectively block bone resorption, the virulence of *Candida*, cancer growth, and metastasis.

## BACKGROUND

### V-ATPases Bind Microfilaments and Other Cytoskeletal Elements

In 1999, vacuolar H^+^-ATPases (V-ATPases) were first shown to bind directly to microfilaments (actin filaments, F-actin) [1]. Data supporting this finding included immunoprecipitation experiments in which F-actin and myosin II were pulled down along with V-ATPase subunits using the monoclonal antibody E11, which binds the E-subunit of V-ATPase. V-ATPase subunits, along with both actin and myosin II, were competitively-inhibited from binding to E11-conjugated beads by the 13 amino acid target peptide of E11. Both actin and myosin II, but not V-ATPase subunits, were removed from immunoprecipitates by depolymerizing microfilaments into actin monomers, suggesting that V-ATPases bind actin filaments directly, and myosin II indirectly by way of microfilaments. This was confirmed by demonstrating that V-ATPases isolated from either mouse osteoclasts or porcine kidneys bound microfilaments composed of actin that had been isolated from rabbit muscles, and which did not contain myosin II.

V-ATPase bound microfilaments with a stoichiometry of 1 V-ATPase bound per every 6-7 actin monomers in a filament, and with an affinity of 55 nM [1]. Transmission electron microscopy of V-ATPases bound to microfilaments showed that they interacted with F-actin through the top of the V_1_ domain Fig. (**1**). This orientation suggested that intact V-ATPases associated with membranes could potentially bind microfilaments. The amount of association between V-ATPases and microfilaments decreased as osteoclasts activated to resorb bone. The changes in binding that were detected biochemically were consistent with the co-localization of F-actin and V-ATPase detected by confocal microscopy. In inactive osteoclasts, there was extensive co-localization between microfilaments and V-ATPases. In resorbing osteoclasts, the amount of co-localization was sharply reduced. A transition state occurred during activation of osteoclasts where both V-ATPases and microfilaments concentrated in a patch together at the site of the nascent ruffled membrane [1]. Later it was shown that a similar transition state occurred as V-ATPase was internalized from the ruffled membrane of activated osteoclasts in response to treatment with phosphatidylinositol 3-kinase (PI 3-kinase) inhibitors [2]. Taken together, these data suggested that binding between V-ATPases and microfilaments might be involved in regulating V-ATPase distribution and/or activity in osteoclasts [1].

Following this initial publication, actin binding sites were identified in both the B1- and B2-isoforms of the B-subunit [2,3], and in the C-subunit [4-6]. The actin binding site in the B-subunit is conserved in organisms as primitive as Baker’s yeast (*Saccharomyces cerevisiae*) [7]. The C-subunit from Tobacco hornworm (*Manducca sexta)* binds F-actin, but the actin binding activity of the C-subunit has not yet been characterized in other organisms [5,6].

Other interactions linking V-ATPases to the cytoskeleton have been described. A PDZ-binding domain was identified at the carboxyl-terminus of the B1-subunit [8]. This interaction domain has been shown to bind sodium-hydrogen exchanger regulatory factor (NHE-RF) [8] and sodium bicarbonate cotransporter (NBC3) [9]. However, the PDZ-domain has not been directly linked to a physiologic function. ADP-ribosylating factor 6 (Arf6) and its activator, ADP-ribosylation factor nucleotide site opener (ARNO), were shown to bind the c- and a-subunits of V-ATPases respectively [10,11]. Arf6 is known to be a vital regulator of both vesicle trafficking and cytoskeletal reorganizations [12-14]. Because recruitment of ARNO was shown to depend on the acidification state of the lumen of the vesicle to which the V-ATPase was attached, it was proposed that V-ATPases could regulate vesicular trafficking and cytoskeletal remodeling in response to pH [10]. V-ATPases have also been shown to interact with several glycolytic enzymes [15-19], which are known to also bind microfilaments [20-24]. Recently, interactions between V-ATPases, fructose bisphosphate aldolase and ARNO were described which may signify the emergence of a mechanism by which the spatial localization and activity of V-ATPases are coupled to the metabolic state of the cell [11].

Based on the accumulated data, Brown and colleagues proposed that, in addition to their enzymatic role as proton pumps, V-ATPases may also be able to coat vesicles and direct the trafficking of the vesicles in the same manner as clathrin, caveolins and coatamer protein complexes [25]. In this scenario, the various interactions with cytoskeletal proteins and cytoskeletal regulators might be required to manage the trafficking of V-ATPase-containing vesicles to their ultimate destinations in cells. Although this hypothesis requires further study, evidence now points to interactions between V-ATPases and cytoskeletal elements as novel targets for drug design. Disruption of key protein-protein interactions might yield unique and cell selective modulators of V-ATPase-dependent functions including bone resorption [26], tissue invasion by cancer cells [27], multidrug resistance [28] and acid-base homeostasis [29]. Unlike traditional inhibitors of enzymatic activity, such inhibitors would function by preventing subsets of V-ATPases from reaching the cellular destinations where they perform cell type specific functions.

Here, we will focus on the direct interaction between V-ATPases and microfilaments that is mediated by the B2-subunit. We will review efforts to understand the function of the microfilament binding site in the B2-subunit, and to develop small molecule inhibitors of the interaction as potential therapeutic agents using a knowledge-based approach. A product of these studies was the identification of enoxacin, a novel inhibitor of osteoclast bone resorption [30]. Efforts are now underway to test the potential of enoxacin and other inhibitors of the B2-microfilament binding interaction for the treatment of bone disease in animal models.

Recently, it was reported that enoxacin is also a selective inhibitor of the virulence of *Candida albicans* [31], and of cancer growth and metastasis [32]. The possible use of enoxacin and related molecules as anti-cancer chemotherapeutic agents emphasizes the need to fully understand the detailed mechanisms by which enoxacin affects cells.

### V-ATPases

Acidification of intracellular compartments is required for a variety of cellular processes such as receptor-mediated endocytosis, protein degradation, and the processing of signaling molecules [33-35]. V-ATPases are large multisubunit enzymes that are expressed at very low levels in most eukaryotic cells and which normally localize to a number of intracellular membranous organelles of the endocytic, exocytic and phagocytic pathways. V-ATPases pump protons across cellular membranes and are critical for the regulation of pH inside intracellular organelles [34]. Because V-ATPases are electrogenic, they also energize membranes. For example, synaptic vesicles require V-ATPases to produce an electrochemical gradient that is utilized to load neurotransmitters [36]. V-ATPases also localize to the plasma membrane of cells such as in renal intercalated cells [37], osteoclasts [29], and metastatic cancer cells [38], in order to carry out cell-type specific functions.

Studies based on cDNA cloning of V- ATPase subunits confirmed the structural and enzymatic relationship that exists between V-ATPases and the mitochondrial F_0_F_1_ ATPase (F-ATPase, ATP synthase) [39]. Thus, much has been inferred about the overall structure of V-ATPases from the still accumulating collection of crystal structures of F-ATPases [40-47]. An even closer structural relationship exists between Archaea V-ATPase (A-ATPase) and eukaryotic V-ATPases [48]. Recently, cryoelectron microscopy studies of V-ATPases, crystallization of individual V-ATPase subunits and crystallization of A-ATPases, have greatly enhanced understanding of the organization of V-ATPases [49-55]. It has become clear that while V-ATPases are evolutionarily-related to, and share important structural design features with F-ATPases and A-ATPases, the three diverge in crucial structural and enzymatic features [56].

V-ATPases are organized into two domains, V_1_ and V_0_ of about^1^ eight and six subunits respectively that operate by a rotary mechanism Fig. (**2**) [57]. The V_1_ domain is an approximately 640 KDa peripheral complex on the cytoplasmic side of the membrane and is responsible for ATP hydrolysis. The alternating heterohexagon of A and B subunits that forms the location where ATP binds and is hydrolyzed, shares high levels of homology between the rotary enzymes. In V-ATPases, the A-subunit is the site of ATP hydrolysis, which is coupled to structural changes in the AB heterohexagon, which powers turning of a central stalk. The A-subunit is closely related to the β-subunit of F-ATPase and B-subunit is a close relative of the α-subunit. The rest of the V-ATPase is less related to F-ATPases, and several of the subunits of the V-ATPases do not have obvious homologs in F-ATPases. Strikingly, V-ATPases likely have three non-identical stator arms, while F-ATPases only have a single stator arm. Each of the stator arms has an EG dimer that interacts with the V_1_ head, but the origins of each in the V_0 _appears to be distinct (Fig. (**2**), large arrows). The V_0_ domain is about 260 KDa and is membrane-embedded. V_0_ mediates proton transport across the membrane [58]. The ATP hydrolysis in the V_1_ domain drives rotation of a ring of c-subunits present in the V_0_ domain, which is coupled to the active transport of protons across the associated membrane [59]. In mammals, the V_0_ domain contains one of four iso-forms of the a-subunit, a large integral protein that is thought to contribute to the proton pore. The a-subunit isoforms, as will be described in more detail later on, are also linked to the sorting of vesicles to different subcellular compartments, although the mechanisms involved in differential sorting are not yet known [60-66].

V-ATPases with the same basic overall structure and enzymatic activity are segregated in cells so that they can perform a variety of housekeeping functions. In some cells, additional subsets exist that perform specialized roles. Osteoclast contain housekeeping subsets of V-ATPases that acidify various cellular compartments [63]. In addition, a specialized subset of V-ATPases is expressed and targeted to the plasma membrane of active osteoclasts [64]. This targeting is particularly interesting because V-ATPases are normally excluded from the plasma membrane.

Many of the V-ATPase subunits exist as multiple isoforms [57]. There are ubiquitous isoforms that are elements of the housekeeping V-ATPases. Some of the ubiquitous isoforms, like B2 in osteoclasts, are also components of specialized V-ATPases [67]. Specialized V-ATPases are typically associated with inclusion of one or more cell and tissue specific isoforms Fig. (**2**). These isoforms are involved in the cell’s specialized function, and are often targeted to cellular domains, like the plasma membrane, beyond those required for the housekeeping functions. For instance, four isoforms of subunit a (a1, a2, a3 and a4) have been identified. Both the a1 and a2 isoforms are ubiquitously expressed. The a3 isoform is highly expressed by osteoclasts [64], microglia [68], and pancreatic beta cells [69], whereas the a4 isoform is highly expressed by renal intercalated cells [65,66] and epididymal clear cells [70]. Mutations of the a3-isoform in humans lead to a condition known as infantile autosomal malignant osteopetrosis, characterized by short, dense, bones that are brittle and more prone to fracture [71,72]. The brittleness is likely due to osteoclasts being unable to remove woven bone to allow its replacement with mature bone during development. Likewise mutations in a4 are associated with a kidney disease called autosomal distal renal tubular acidosis [65].

In eukaryotic cells, very precise spatiotemporal targeting of V-ATPases is required for cell survival. Despite the fundamental importance of this regulation, it is not understood mechanistically. The precision by which V-ATPases must be delivered to specific cellular compartments would seem to require direct or indirect associations with cytoskeletal elements.

### Subcellular Localization of V-ATPase During Osteoclast Activation

Osteoclasts are specialized cells that invade mineralized tissue in a highly regulated manner [73]. Invasion requires degradation of both the organic and inorganic elements of bone. When osteoclasts contact mineralized bone under permissive regulatory conditions, they polarize, forming a specialized resorptive structure called the ruffled plasma membrane (ruffled membrane, ruffled border), which is encircled by an equally unusual cytoskeletal structure called the actin ring Fig. (**3**) [26,74].

The actin ring is formed as a coalescence of distinct structures called podosomes [74,75]. These, or very similar, structures also exist in other cells that migrate through tissue [76,77]. This includes metastatic tumor cells [78]. In osteoclasts, V-ATPases acidify the extracellular space between the ruffled border and bone surface [79]. The low pH dissolves the bone mineral and provides an environment in which the acid cysteine proteinase, cathepsin K, can digest the organic components of the bone [80, 81].

During osteoclast differentiation, the levels of most V-ATPase subunits increase and cell-specific isoforms are expressed (a3, d2) leading to increased levels of a subset of V-ATPases with an unusual subunit composition [82]. This subset of specialized V-ATPases is stored within intracellular vesicles [26]. The ruffled membrane is a highly convoluted membrane that forms as the V-ATPase-packed vesicles are directed to the site of the nascent ruffled plasma membrane in coordination with actin filaments, and then fuse with the plasma membrane [1]. Once bone resorption is completed, the V-ATPases are re-internalized into the cytoplasm and the cell can then move to another site of resorption and reform actin rings and ruffled membranes for another round of resorption [2,83].

### Characterization of V-ATPase Binding to Microfilaments Through the B-Subunit

After the initial identification of a direct interaction between V-ATPases and microfilaments [1], the next challenge was to identify the subunit or subunits that mediated the interaction. Blot overlay studies in which F-actin was used to probe V-ATPases that had been isolated, separated by SDS-PAGE and transferred to nitrocellulose, identified B-subunit as a potential F-actin binding protein [3]. This was supported by studies in which isolated V-ATPases were selectively disassembled to eliminate most of the subunits except the B-subunit, which still bound F-actin. Bacterially-expressed recombinant fusion proteins containing various fragments of the B1 and B2 subunits demonstrated that fragments as small as amino acids 23-67 of the B1 subunit and 29-73 of B2 bind microfilaments as tightly as full length subunits, but smaller fragments did not bind [3]. The fusion proteins bound F-actin with a stoichiometry of one fusion protein per one actin subunit and with apparent dissociation constants of between 100-200 nM for both isoforms. Later, it was reported that B-subunit from *Manducca sexta* bound F-actin [84] and B-subunit from yeast [7] bound actin with similar affinity to mammalian B-subunits. These data suggested that the capacity of B-subunits of V-ATPase to bind microfilaments developed very early in evolution and has been conserved. Both B2, the ubiquitously-expressed B subunit in mammals, which is expressed at high levels in osteoclasts, and B1, which is restricted to epithelial cells in the kidney and a few other tissues where V-ATPases are targeted to the plasma membrane, bound actin equally well. This indicates that the actin binding activity of B-subunits may have a role in various cellular processes. Even though B1 and B2 bound F-actin equally well, the sequence of the actin binding region varied considerably [2]. Yeast and *Manduca* only express one B-subunit. The actin binding domain of yeast and the putative binding domain in *Manduca *are more homologous to B2 than B1.

Because B-subunits are intimately involved in the enzymatic function of housekeeping V-ATPases, in order to understand the function of the actin binding activity it was vital to identify amino acids in B-subunits that are crucial for actin binding activity, but not for the V-ATPase’s enzymatic activity. During the mapping and characterization of the actin-binding region on B subunits, a 13 amino acid sequence similar to a portion of the actin-binding site of mammalian profilin was identified by direct inspection of the sequence by Dr. Michael R. Bubb, (North Florida/South Georgia Veterans Health System, Research Service, Gainesville, Florida)[2]. Studies of synthesized peptides confirmed that this profilin-like sequence had the capacity to bind actin. Peptides derived from the actin-binding site on the B-subunit competed with profilin for binding to actin. Moreover, point mutations known to disrupt the actin-binding activity of profilin [85, 86, 87], also decreased the actin-binding activity of the B-subunit derived peptides [2]. These data indicated that the profilin-like region is a vital element of the actin binding domain of B-subunits [2].

Genetic alteration of the sequence of recombinant B1 and B2, by replacing the profilin-like domain with an identical length spacer composed of the sequence of the B-subunit from the Archaean, *Pyrococcus horikoshii*, eliminated actin binding activity [2]. We had chosen the use of this spacer reasoning that it would probably not alter the overall structure of the subunit, and that because Archaeans do not have a microfilament–based cytoskeleton, it would be unlikely for them to have actin binding activity. We had also found that there were specific differences in the profilin-like domain amino acid sequence in *Pyrococcus *that reduced the capacity of synthetic peptides to bind actin. We later confirmed in a yeast model that substitution of the “profilin-like” domain with the spacer did not affect the enzymatic activity of the V-ATPase, even though it eliminated the ability of the pump to bind microfilaments [7]. This provided the opportunity to test the physiologic role of the actin binding activity of B-subunit.

### Genetic Analysis of the Physiologic Role of the Actin Binding Activity of B-Subunits

The yeast, *Saccaromyces cerveciae*, contains V-ATPases that are composed of subunits that are very similar in sequence to those found in mammals [88]. Unlike most other eukaryotic cells, these yeast do not require V-ATPase enzymatic activity [88]. As long as they are maintained under acidic culture conditions, they can internalize and make use of sufficient protons for housekeeping functions. However at alkaline external pH, yeast requires V-ATPase activity. This allows replacement of wild type subunits with mutants [89-92].

The actin binding activity of B-subunit was eliminated by introduction of a B-subunit in which the profilin-like domain was replaced with the Archaea-derived spacer as described above [7]. Wild-type B-subunit was also introduced to B-subunit knockouts in parallel. This mutant did not affect the ability of the yeast to survive under normal culture conditions in alkaline pH. However, yeast containing the mutant construct grew two orders of magnitude slower in the presence of sub lethal doses of wortmannin and cyclohexamide compared with yeast expressing wild type B-subunits. This suggests that the actin binding activity in B-subunit may have initially evolved as an element of a response to environmental toxins.

To test the role of the actin binding activity of B-subunit in osteoclasts, simultaneous deletion of endogenous B2-subunit and replacement with mutant B2 was not feasible. However, it had been shown that when the same cell expresses both the B2 and B1 subunits, the two isoforms do not assemble into the same V-ATPase [93]. This allowed analysis of exogenously introduced wild type B1, or B1 lacking actin binding activity (B1mut) on a background of normal osteoclast activation. To deliver B1 and B1mut adeno-associated virus serotype 2 vectors were used [94]. Both B1 and B1mut assembled with other V-ATPase subunits in osteoclasts, and both localized to vesicular compartments in inactive osteoclasts. B1 was efficiently targeted to the ruffled plasma membrane of resorbing osteoclasts in the same manner as the endogenous B2 subunit. However, B1mut was never detected in ruffled plasma membranes [94]. These results suggested that the actin binding activity of B-subunit is necessary to traffic V-ATPases to vesicles that later fuse with the plasma membrane to form the ruffled plasma membrane. Therefore, an inhibitor of the interaction between the B2-subunit and microfilaments would be expected to inhibit bone resorption Fig. (**4**).

### Inhibition of the Binding Interaction between V-ATPase and Microfilaments as a Mechanism for Osteoclast Regulation 

To screen for small molecule inhibitors of the B2-microfilaments interaction, we made use of two tools. First, even though B2 has not been crystallized, we were able to construct a virtual high confidence atomic level model of the actin-binding domain of B2 [30]. We made use of the close sequence homology between the α-subunit of F-ATPase (for which many crystal structures are available) and B2 as the primary guide in modeling the B2 structure. In addition, the structure was informed by the relationship between B2 and profilin. Profilin had been crystallized in complex with actin, and this allowed better insight into the actin-binding surface of B2 [95].

Second, computer-based virtual screening at the very best reduces the number of candidate molecules from 100,000’s to 100’s [96-98]. It is still vital to have a reliable medium throughput activity assay, ideally using pure proteins. We made use of a simple microfilament pelleting assay to determine whether 100 µM of each of the test molecules affected interaction between recombinant B2 and rabbit muscle F-actin [30]. From the top ranked 40 molecules identified in a virtual screen of over 300,000 small molecules in a library from the National Cancer Institute, we found that 4 strongly inhibited the interaction between B2 and F-actin in the pelleting assay. Of these, two were found to inhibit osteoclast formation and activity with an IC_50_ around 10 µM without affecting the viability of the cells, while the other two killed osteoclasts and other cells. The lethal molecules had been identified in previous screens and were known to be toxic to cells for reasons that were unrelated to the V-ATPase.

To date we have focused our attention primarily on enoxacin, which is a second generation fluoroquinolone antibiotic [99]. It was used in the United States for about ten years for the treatment of urinary tract infections and gonorrhea, and was voluntarily taken from the market in the US because of adverse effects including insomnia [100]. It is still used in much of the rest of the world as an antibiotic. While side effects have been described in humans, they are relatively rare and mild. It is plausible that some of the side effects linked to enoxacin such as: insomnia, dizziness, and photosensitivity might be the result of disrupting the B2-microfilament interaction in other cell types. For example, we have reported that V-ATPase-microfilament interactions occur in microglia [68]. More studies will be required to identify other cells that might be affected by inhibitors of B2-microfilament interactions and subsequent effects. Other side effects associated with enoxacin, for instance: tendon ruptures, are likely class-action effects of quinolones [101] since they have also been linked to fluoroquinolones that do not affect the microfilament-B2 interaction [30]. A practical advantage to studying enoxacin is that it can be obtained in large quantities for modest prices making detailed *in vitro* and *in vivo* studies possible.

Enoxacin and the other inhibitors identified blocked the formation of multinuclear cells that are positive for tartrate-resistant acid phosphatase activity (TRAP+)[30]. The IC_50_ for inhibition of osteoclast formation was approximately 10 µM. The phenotype was somewhat surprising to us because knockout of the a3-subunit of V-ATPase results in osteoclasts that fuse and express TRAP activity normally, but which fail to transport V-ATPases to the plasma membrane when osteoclasts encounter activation signals [64]. However, mutations in the d2-subunit of V-ATPase result in a phenotype of osteoclasts, including reduced TRAP expression and decreased fusion of precursors [102], that resembles the effects of enoxacin. The underlying mechanisms that result in the phenotype observed in the osteoclasts in which d2 is mutated are not yet understood but seem to involve failure to express a disintegrin and metalloproteinase domain-containing protein (ADAM) 8 and ADAM12 normally. Efforts are underway to determine whether the mechanisms underlying the phenotype of enoxacin-treated cells are identical to those observed in d2-knockout osteoclasts.

We also showed that if osteoclasts were treated with enoxacin after they have differentiated into TRAP+ multinuclear cells and loaded onto bone slices, a reduction in bone resorption, formation of actin rings as well as the transport of V-ATPases to the plasma membrane to form ruffled membranes was observed [30]. Similarly, the IC_50_ was approximately 10 µM. Studies are underway to test the hypothesis that these effects are due to inhibition of the interaction between the B2-subunit and microfilaments, and if so, to determine the mechanism.

Importantly, enoxacin did not affect osteoblast growth and survival, or the ability of osteoblasts to mineralize at concentrations as high as 100 µM [30]. This showed that enoxacin is selectively active against osteoclast bone resorption. The basis of this selectivity may be because osteoblasts, like most cells, do not display V-ATPases bound to microfilaments. We are currently seeking to understand the reason why the actin-binding of the B2 subunit is not made use of in osteoblasts and other cells (see below for more extensive discussion of this question).

Because enoxacin acts by a different mechanism than current inhibitors of osteoclasts that are used therapeutically (bisphosphonates, denosumab) we hypothesize that enoxacin and like molecules may have advantages. Specifically, because enoxacin does not kill osteoclasts like bisphosphonates, or prevent signaling leading to osteoclast differentiation like denosumab [103], we suspect that the treated cells may continue to produce regulatory signals necessary to recruit and activate osteoblasts [104] and that enoxacin and like molecules may be bone anabolic. This would be predicted if the effects of enoxacin have the same mechanistic underpinnings as those detected in d2-knockout osteoclasts [102].

### Key Questions for the Future

#### Regulation of B-Subunit Binding to Microfilaments

Based on the empirical finding that V-ATPases derived from osteoclasts, but not most other cell types, are associated with the actin cytoskeleton, we hypothesized that this interaction is selectively important for osteoclast function. Our data so far has supported this idea. However, the regulatory mechanism by which the B2 binding interaction with F-actin remains unexplained. We tested whether the interaction might be directly regulated by signaling phosphoinositides since the actin binding site in B-subunit is related to part of the actin binding site in profilin, and profilin is well known to be regulated by phosphatidylinositol 4,5-biphosphate (PIP2) [105]. In addition, we found that we could modulate the amount of V-ATPase bound to microfilaments recovered in osteoclasts using phosphatidylinositol 3-kinase inhibitors [2]. These inhibitors also triggered rapid internalization of V-ATPases from the ruffled membranes of actively resorbing osteoclasts. Based on these data, we examined whether PIP2 or other phosphatidylinositol phosphates directly inhibited the interaction between B2- and microfilaments but, so far, we have been unable to detect such regulation in our *in vitro *binding assays.

Recent structural data indicate an alternative mechanism for the regulation of the B-subunit-microfilament interaction. Direct visualization of V-ATPase by cryoelectron microscopy and crosslinking studies support the concept that the EG portion of the three stator arms are likely to be physically blocking the actin binding site of B-subunit [54,106,107]. If so, for a B-subunit within a V-ATPase to bind F-actin, at least one of the EG dimers must be absent. One simple hypothesis would be that certain a-subunit isoforms (perhaps a3 and a4) do not support assembly of a stator arm, leaving the actin binding site on one B-subunit in the heterohexamer of AB free to bind actin. This hypothesis predicts that in osteoclasts, only the a3-subunit containing V-ATPases would be bound to microfilaments, and that these V-ATPases would only have two stator arms and two EG dimers per enzyme. Efforts are underway in our lab to test this idea. A corollary to that hypothesis is that the three EG containing “stators” may be required for regulation and not for the enzymatic function of the proton pumps. Muench and colleagues, suggested from biophysical and structural arguments that only two stators are required by a proton pump and therefore it is a distinct possibility that the third stator may be present to allow more regulatory complexity [107].

#### Inhibition of Osteoclast Formation by Enoxacin

If enoxacin and other inhibitors act due to blocking the B2-subunits binding to microfilaments, then the resulting phenotype of the osteoclasts resembles that of d2- knockout osteoclasts, which also have reduced fusion and TRAP activity. These observations clearly require explanation. The authors of the article describing d2-knockout osteoclasts proposed that d2 might have a secondary role as fusagen, and reported that the cell fusion-associated proteases ADAM8 and ADAM12 mRNA levels were reduced in the d2-knockout [102]. In addition, overexpression of exogenous ADAM8 and ADAM12 in d2-knockout osteoclasts partly restored their ability to fuse. We are examining levels of ADAM8 and ADAM12 after treatment with enoxacin. A mechanism by which d2 might serve as a fusagen has not yet been described, but d2 appears to be expressed at normal levels after treatment with enoxacin.

Concurrent with our identification of enoxacin as an inhibitor of B2-microfilament binding, another group screening for small molecule modulators of microRNA activity identified enoxacin as a stimulator of microRNA processing, and suggested this was through interaction with TAR RNA-binding protein 2 (TRBP2) [108]. This finding could, in principle, explain some or all of the observations with regard to osteoclast formation and function. MicroRNAs are short 21-23 nucleotide, non-coding RNAs that bind to target sequence on specific mRNAs and prevent those mRNAs from being translated [109-112]. We found that fluoroquinolones that were reported to stimulate microRNA activity almost as well as enoxacin, had no effect on osteoclasts, and conversely, that pefloxacin, which does not stimulate microRNA activity [108], inhibited the V-ATPase-microfilament interaction, and osteoclast formation and function (though not quite as well as enoxacin) [30]. Moreover, we have identified another small molecule inhibitor of the B2-microfilament interaction that has a completely different structure, but which inhibits osteoclast differentiation and bone resorption in the same way as enoxacin (Holliday, Ostrov, Wronski, Unpublished data). It would seem very unlikely that this small molecule also affects microRNA regulation like enoxacin in addition to blocking the B2-microfilament interaction, unless changes in microRNAs are downstream of inhibition of the V-ATPase-microfilament interaction.

### Enoxacin and Cancer

Recently, Melo and colleagues reported that “enoxacin is a cancer-specific [sic] growth inhibitor that acts by TRBP2-mediated microRNA processing”[32]. In this exciting study, the authors showed that enoxacin inhibits a variety of human cancers in tissue culture, although the IC_50_ was 124 μM. More impressively, 10 mg/kg enoxacin injected daily (a dose only slightly higher than that used to treat infections) dramatically-reduced both the growth and metastasis of two different human colorectal cancers in a nude mouse model. Also, evidence was presented that Tar-RNA binding protein 2 (TRBP2) is involved in the mechanism by which the colorectal cells were inhibited.

It was not reported whether inhibition of the B2- microfilament interaction played any role in the response of cancer cells to enoxacin. This should be examined in the future given that plasma membrane V-ATPases have been reported in metastatic tumor cells [27], and the a3- and a4-subunits are expressed in at least some cancer cells [38]. It is therefore plausible that disruption of the interaction between B2 and microfilaments by enoxacin plays a role in the inhibition of cancer that was described.

Within the context of cancer, it is of interest that enoxacin disrupted actin rings in osteoclasts [30]. As described earlier, actin rings are composed of structures called podosomes, which are similar or identical to podosomes (also called invadopodia) in metastatic cancer cells that are important for tissue invasion [113]. It will be of interest to determine whether enoxacin inhibits the formation of podosomes in cancer cells.

### Enoxacin and *Candida*

Yeast infections are a very serious medical problem for individuals that are immunocompromised [114]. Enoxacin was identified as an inhibitor of the virulence of *Candida* in a screen of small molecules with no known anti-fungal properties [31]. The B-subunit of the yeast *Saccharomyces cerevisiae *was shown to bind microfilaments and its actin binding activity was demonstrated to be important for the survival of yeast in the presence of sublethal doses of specific drugs [115]. The B-subunit of *Candida albicans* is very similar to *Saccharomyces cerevisiae* and to the mammalian B2-subunit in the actin binding region, and key residues known to be important for actin binding activity are conserved. Although further studies will be required, it is possible that the effects of enoxacin on the virulence of *Candida* are due to disrupting B-subunit microfilament binding.

### Perspective on the Rational Identification of V-ATPase-Directed Therapeutics 

V-ATPase has both housekeeping roles and specialized roles in specific cell types that are related to important human pathologies including cancer and bone disease [34]. Significant efforts have been put forth attempting to identify inhibitors of V-ATPase enzymatic activity that are selective for subsets of V-ATPases [116,117]. To date this work has not yielded therapeutic agents that are useful in the clinic. We demonstrate that it is possible to make use of emerging computational chemistry approaches along with data gleaned from cell and molecular studies, to identify novel types of V-ATPase-directed osteoclast inhibitors. We expect our current and future studies to determine whether enoxacin or other inhibitors of the interaction between B2- and microfilaments prove useful for the treatment of human pathologies. In addition, the basic strategy we have taken should be applicable to other types of V-ATPase-directed inhibitors. However, such work will require better structural understanding of V-ATPases and better characterization of the binding sites for interacting proteins like Arf6, ARNO and aldolase, and the C-subunit binding sites for microfilaments. It also seems likely that there may be additional interactions between V-ATPase subunits and interacting proteins that have yet to be identified and which may offer potential therapeutic targets.

More generally, the studies described involve targeting specific and specialized interactions of a housekeeping enzyme. The goal of the final product is not to kill the cells, but to regulate cell–type selective machinery so that there is less of an undesired activity. There may be many new therapeutic opportunities with this approach, but it will likely require using assays that are not amenable to high throughput screens. A better understanding of how protein-protein interactions define cell-type specific functions and of the structural basis for these interactions at the atomic level would make increasingly more possible to make use of “virtual screens” as an alternative to high throughput screens, and as a practical shortcut in the drug discovery process [118-123]. For this to occur, computational chemistry must prove itself in the real world. Our studies represent one out of a number of examples that have emerged during the past few years showing the feasibility of knowledge-based virtual screening as a rational approach to drug discovery [30, 124-128]. However, there still is considerable skepticism. Until the reliability of these approaches is better documented and more widely accepted, it is possible that exploration of many fruitful drug development opportunities will be delayed.

## Figures and Tables

**Fig. (1):**
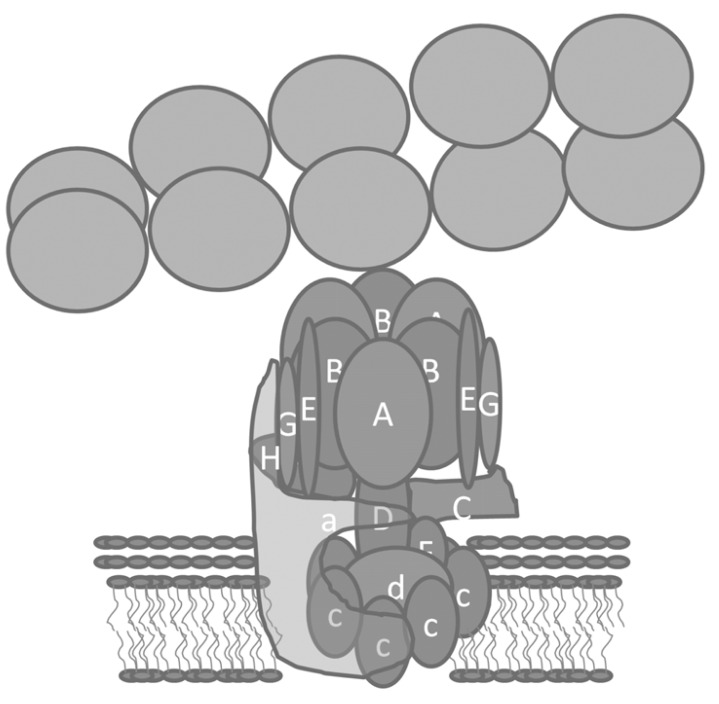
**Microfilament interacting with V-ATPase through the B-subunit.** Note that because of the location of the F-actin binding site, V-ATPases in a membrane can bind microfilaments. Arbitrarily, one of the EG stator arms has been removed to allow access of microfilaments to the actin binding site on the B-subunit. In contrast, to B-subunit, the actin binding sites in the C-subunit are unlikely to be accessible in the intact enzyme and instead the interaction between C-subunit and actin may be involved in reversible assembly [128].

**Fig. (2):**
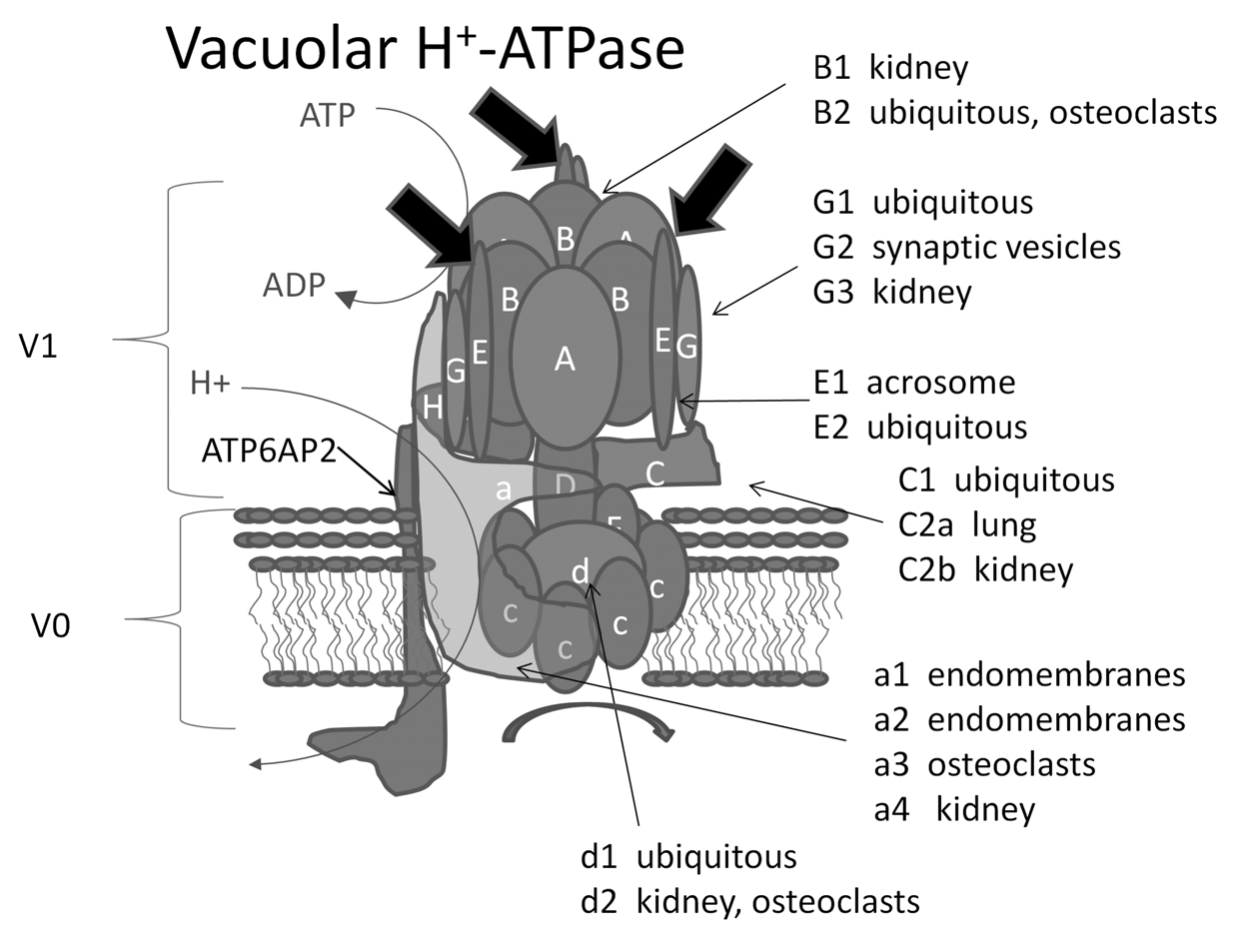
**Structure of V-ATPase.** Structure is based on the recent electron microscopy study by Harrison and colleagues (53). List of subunit isoforms was adapted from reference 33. Curved thin arrows indicate site of ATP hydrolysis and the pathway of protons through the membrane. The fat curved arrow shows the direction of turning of the rotor during enzymatic activity. Large straight arrows identify the three non-identical EG stators. ATP6AP2 (the Pro-renin receptor) is included in the schematic although its location is not known, nor whether it is associated with all V-ATPases or only with V-ATPases in specific locations. Adapted from (Neubert, J. K., R. M. Caudle, C. Dolce, E. J. Toro, Y. Bokrand-Donatelli, and L. S. Holliday. 2011. Neural modulation of orthodontic tooth movement. In *Orthodontics* (Mandic, ed), INTECH, Open Access Publisher, Rijeka, Croatia In press) with permission.

**Fig. (3):**
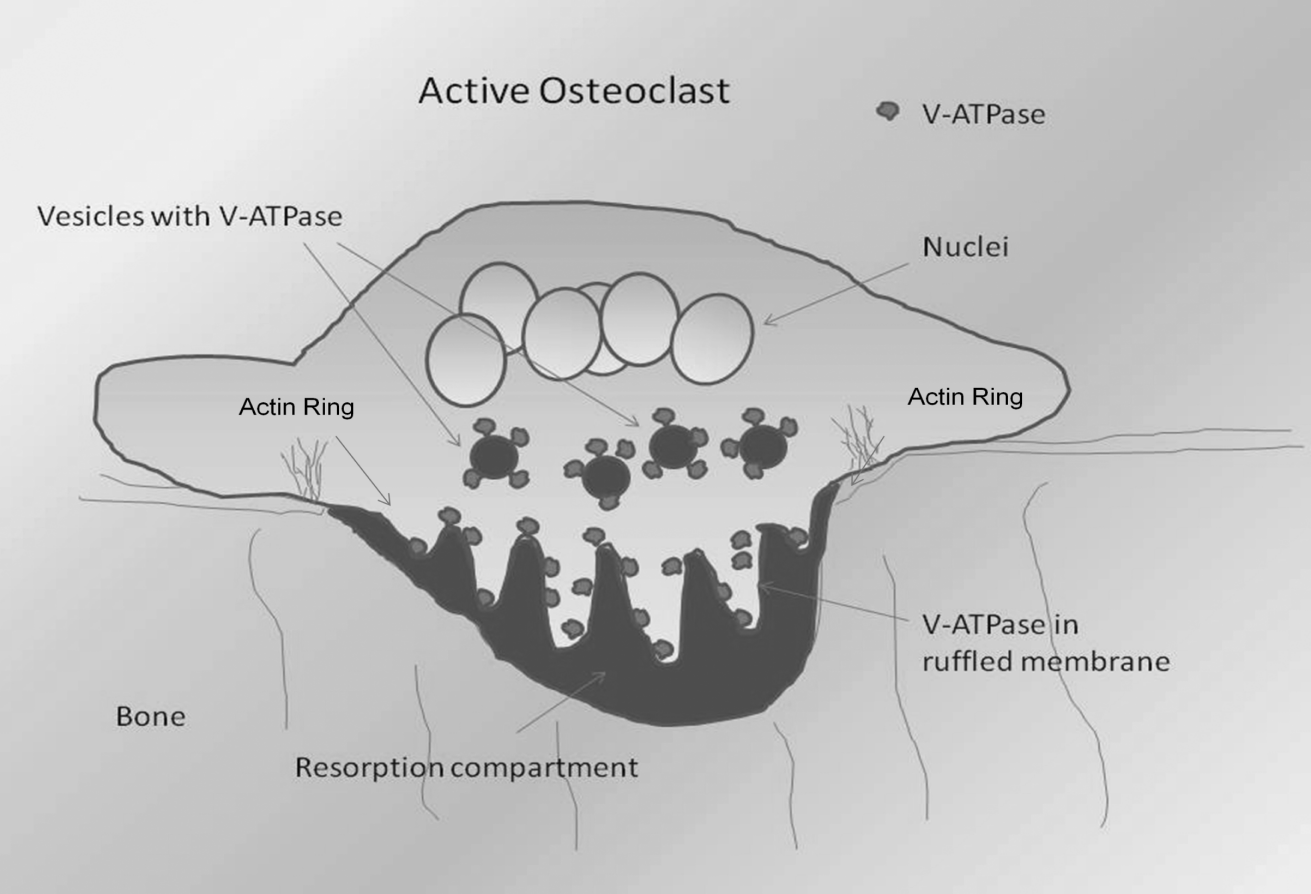
**Organization of resorbing osteoclast.** The schematic shows a side view of a resorbing osteoclast and depicts important features required for resorption. V-ATPases in vesicles are targeted to the the ruffled plasma membrane, a subdomain of the plasma membrane. The ruffled membrane is bounded by an actin ring which is composed of podosomes. The actin ring forms concurrent with the insertion of V-ATPases into the plasma membrane. The actin ring is associated with a sealing zone, a region of very tight adhesion that segregates an extracellular resorption compartment. V-ATPases pump protons into the resorption compartment lowering its pH which solubilizes bone mineral, and allows the acid cysteine proteinase, cathepsin K to degrade the organic matrix of bone. Adapted from (Neubert, J. K., R. M. Caudle, C. Dolce, E. J. Toro, Y. Bokrand-Donatelli, and L. S. Holliday. 2011. Neural modulation of orthodontic tooth movement. In *Orthodontics* (Mandic, ed), INTECH, Open Access Publisher, Rijeka, Croatia In press) with permission.

**Fig. (4):**
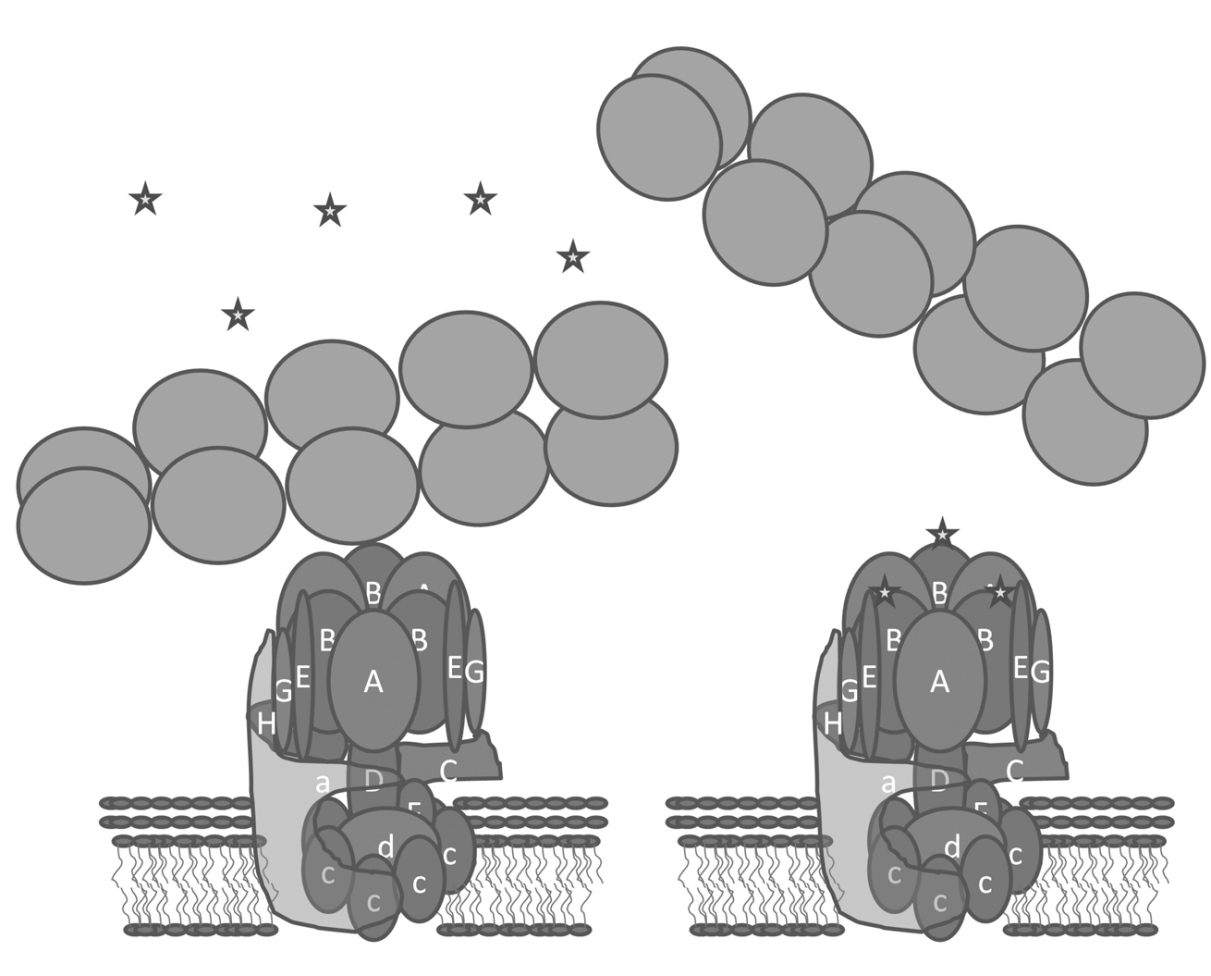
**Small molecule inhibitors of V-ATPase binding to microfilaments.** We hypothesized that small molecules (depicted as stars) that bind the actin binding surface on B-subunit would competitively inhibit binding to F-actin and inhibit osteoclast bone resorption.

## Footnotes

1 V-ATPases from different organisms, and perhaps different tissues within the same organism, contain different numbers of subunits. For example, some organisms have 3 c-subunits (c, c’,c”) but this is not universal. The nature and role of the accessory proteins Ac45 and the prorenin receptor requires clarification. These will be discussed in detail in another review within this issue. It is also worth noting that the consensus structure and subunit composition of the V-ATPase has evolved considerably in the past few years as better techniques have developed for their analysis, and further adjustments may be required in the future when crystal structures appear.
